# Impact of the COVID-19 pandemic on tuberculosis national reference laboratory services in the WHO European Region, March to November 2020

**DOI:** 10.2807/1560-7917.ES.2021.26.24.2100426

**Published:** 2021-06-17

**Authors:** Florian P Maurer, Natalia Shubladze, Gulmira Kalmambetova, Irina Felker, Giorgi Kuchukhidze, Francis Drobniewski, Askar Yedilbayev, Soudeh Ehsani, The European Laboratory Initiative on TB, HIV and Viral Hepatitis, Additional members of the European Laboratory Initiative on TB, HIV and Viral Hepatitis core group (in alphabetical order), Additional members of the European Laboratory Initiative on TB, HIV and Viral Hepatitis core group (in alphabetical order), Ana Avellón, Zamira Baydulloeva, Vladimir Chulanov, Daniela Maria Cirillo, Dmitry Kireev, Claudio U Köser, Stefan Niemann, Ecaterina Noroc, Roger Paredes, Rob Schuurman, Elina V Sevastyanova, Daniel Simões, Alena Skrahina, Maja Stanojevic.

**Affiliations:** 1 National and WHO Supranational Reference Laboratory for Mycobacteria, Research Center Borstel, Leibniz Lung Center, Borstel, Germany; 2 Institute of Medical Microbiology, Virology and Hygiene, University Medical Center Hamburg-Eppendorf, Hamburg, Germany; 3 National Reference Laboratory, National Center for Tuberculosis and Lung Diseases, Tbilisi, Georgia; 4 National TB Reference Laboratory, Bishkek, Kyrgyzstan; 5 Scientific department, Novosibirsk Tuberculosis Research Institute, Novosibirsk, Russia; 6 Regional Office for Europe, World Health Organization, Copenhagen, Denmark; 7 Infectious Diseases, Faculty of Medicine, Imperial College London, London, United Kingdom; 8 The named authors are members of the European Laboratory Initiative on TB, HIV and Viral Hepatitis core group. The additional members of the European Laboratory Initiative on TB, HIV and Viral Hepatitis core group are listed under Investigators

**Keywords:** SARS-CoV-2, diagnostics, PCR

## Abstract

We assessed the impact of COVID-19 on diagnostic services for tuberculosis (TB) by national reference laboratories in the WHO European Region. Of 35 laboratories, 30 reported declines in TB sample numbers, amounting up to > 50% of the pre-COVID-19 volumes. Sixteen reported reagent or consumable shortages. Nineteen reallocated ressources to SARS-CoV-2 testing, resulting in an overall increase in workload, largely without a concomitant increase in personnel (n = 14). This poses a risk to meeting the 2025 milestones of the End TB Strategy.

The ongoing coronavirus disease (COVID-19) pandemic presents a notable challenge to healthcare systems worldwide. A survey conducted within the European reference laboratory network for tuberculosis (TB), which comprises 31 national TB reference laboratories from the European Union / European Economic Area (EU/EEA) Member States and the United Kingdom, found a substantial impact of the pandemic on TB reference services between March and June 2020. Specifically, training and research activities, sample turnaround times, access to external quality assessment, and the availability of selected diagnostic services were affected [1]. Moreover, the COVID-19 pandemic had a considerable impact on testing for HIV, viral hepatitis, and sexually transmitted infections in the World Health Organization European Region [2].

Here, we set out to expand these early observations on the the impact on TB diagnostic services in the WHO European Region from March to November 2020, with a focus on countries with a high burden of multidrug-resistant TB (MDR-TB), defined as being resistant to rifampicin and isoniazid.

## Capturing the state of reference laboratory services March to November 2020

An online survey, jointly prepared by the European Laboratory Initiative (ELI) and the WHO Regional Office for Europe Joint TB, HIV and Viral Hepatitis (JTH) programme, was conducted among directors of TB NRL from all countries within the Region. Nine questions addressed the quantitative impact of the pandemic on TB sample numbers, shortages in reagents and consumables, as well as reallocation of TB laboratory infrastructure or staff to severe acute respiratory syndrome coronavirus 2 (SARS-CoV-2) testing. Eligible participants were invited by ELI/JTH to complete the survey between December 2020 and April 2021. Participants were blinded towards responses from other laboratories throughout the survey. Results were collected electronically and analysed by ELI/JTH.

**Figure 1:**
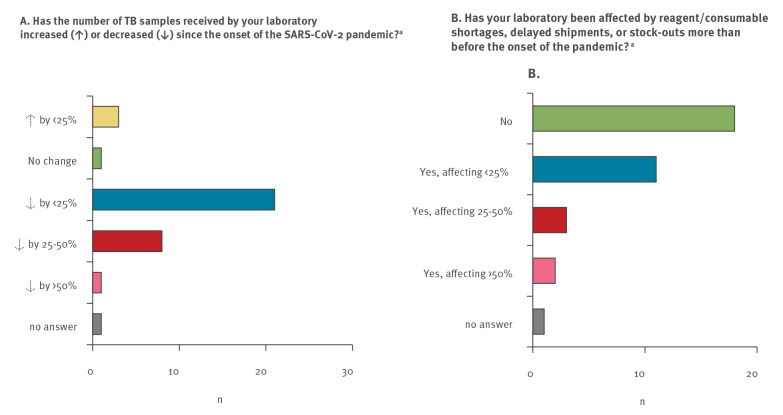
Impact of the COVID-19 pandemic on tuberculosis diagnostic services at 35 tuberculosis national reference laboratories, WHO European Region, March−November 2020 (n = 33 countries)

COVID-19: coronavirus disease; SARS-CoV-2: severe acute respiratory syndrome coronavirus 2; TB: tuberculosis; WHO: World Health Organization.

^a ^34 responses.

In total, 35 of 44 eligible laboratories responded to the survey. The laboratories were located in Albania, Armenia, Azerbaijan, Belarus, Belgium, Bosnia and Herzegovina, Bulgaria, Croatia, Denmark, Estonia, Finland, France, Georgia, Germany, Hungary, Ireland, Italy, Kazakhstan, Kyrgyzstan, Latvia, Moldova, Montenegro, the Netherlands, Poland, Portugal, Romania, Russia (three eligible laboratories), Slovenia, Spain, Sweden, Tajikistan, Turkey, and Uzbekistan. They included 10 reference laboratories from eight of the nine high MDR-TB burden countries in the WHO European Region, comprising the three eligible reference laboratories from Russia [3]. Thirty NRL reported declines in sample numbers received for TB testing ranging from < 25% (n = 21), and 25–50% (8), to > 50% (n = 1) compared with the average pre-COVID-19 testing volume (Figure 1). Declines in sample numbers submitted to the 10 NRL from countries with a high burden of MDR-TB were mostly reported to be < 25% (n = 6). Two laboratories reported declines of 25–50%, and two laboratories reported no decline.

Almost half (n = 16) of the participating laboratories, including three of 10 laboratories from high MDR-TB burden countries, reported to have been affected by reagent or consumable shortages, delayed shipments, or stockouts (Figure 1). Two laboratories reported shortages of > 50% of reagents or consumables.

## SARS-CoV-2 testing in tuberculosis reference laboratories

**Figure 2:**
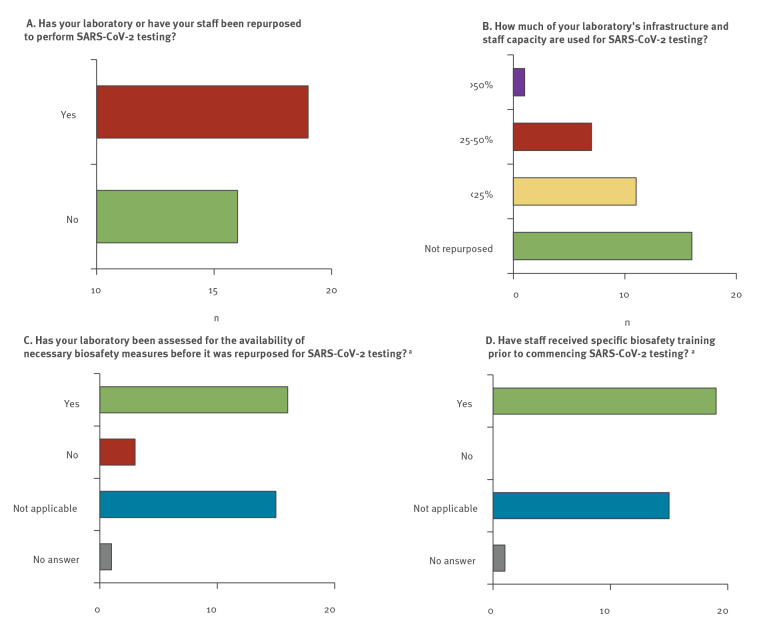
Implementation of SARS-CoV-2 testing at 35 tuberculosis national reference laboratories in the WHO European Region, March−November 2020 (n = 33 countries)

SARS-CoV-2: severe acute respiratory syndrome coronavirus 2; WHO: World Health Organization.

^a^ 34 responses.

TB diagnostic infrastructures offer high spatial coverage, preexisting supply chains, staff trained to work with airborne pathogens, and the availability of analytical and biosafety equipment. Leveraging the potential of TB laboratories for SARS-CoV-2 testing has therefore been an evident consideration in the laboratory response to the COVID-19 pandemic, particularly in otherwise resource-poor settings. Respective guidance was issued by WHO, the Stop TB Partnership, and others [4-7]. In our survey, 19 of 35 participating laboratories reported having reallocated infrastructure and/or workforce to SARS-CoV-2 testing, including five of 10 laboratories from countries with a high burden of MDR-TB (Figure 2). The extent to which laboratory infrastructure or staff were reassigned to the COVID-19 response varied between < 25% (n = 11), 25–50% (n = 7), and > 50% (n = 1) (Figure 2). Sixteen of the 19 laboratories that reallocated ressources to the COVID-19 response confirmed that they had performed laboratory biosafety assessments before handling SARS-CoV-2 samples, and all 19 laboratories reported having conducted specific biosafety training with the involved staff (Figure 2).

**Figure 3:**
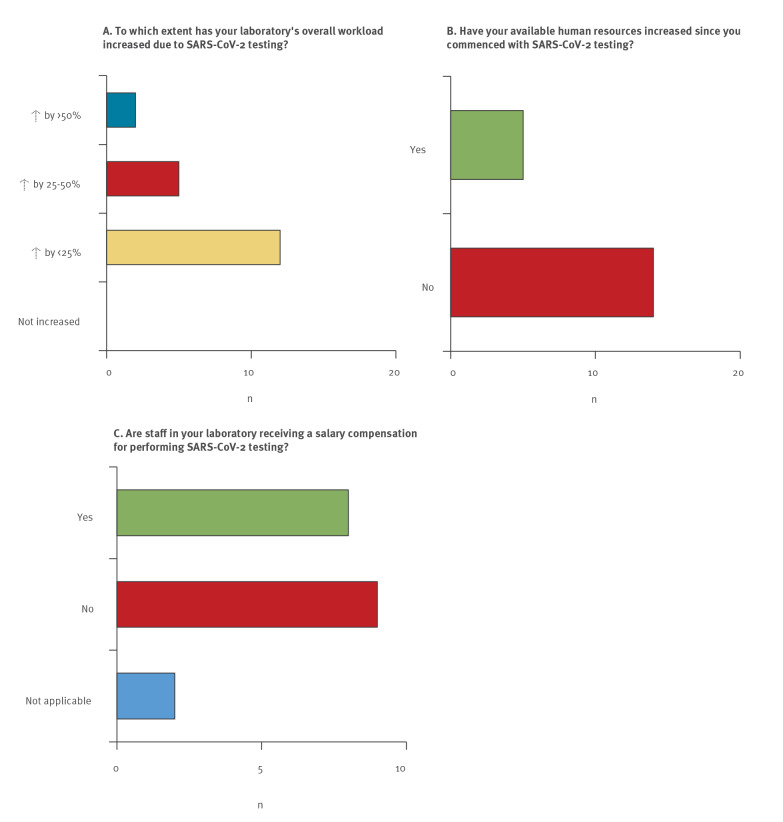
Impact of SARS-CoV-2 testing on overall workload in 19 tuberculosis national reference laboratories (n = 17 countries)

Notably, while a large majority of participants reported declines in sample numbers sent for TB diagnostics, the overall workload in the 19 laboratories that reallocated ressources to SARS-CoV-2 testing increased by between < 25% (n = 12), 25–50% (n = 5), and > 50% (n = 2) (Figure 3). Only five of the 19 laboratories involved in the COVID-19 response reported increases in personnel and just eight laboratories reported salary compensations for staff performing SARS-CoV-2 testing in addition to their regular duties (Figure 3). The two laboratories that reported the highest increases in the overall workload were located in countries with a total annual TB incidence rate well above the average of the WHO European Region, pointing to a substantial workload for both diseases at the same time. One of these laboratories reported granting additional payments for the involved personnel but no staff increases, while the other reported the availability of additional human ressources without salary compensations.

SARS-CoV-2: severe acute respiratory syndrome coronavirus 2.

Only the 19 responses by laboratories that indicated performing SARS-CoV-2 testing in question ‘Has your laboratory or have your staff been repurposed to perform SARS-CoV-2 testing?’ (Figure 2 panel A) are shown.

## Discussion

Before the start of the COVID-19 pandemic, TB was the leading cause of death because of a single infectious agent, affecting 10 million people in 2019 and causing 1.4 millon deaths worldwide [3]. With an overall percentage reduction in the TB incidence rate (new and relapse cases per 100,000 population per year) of 19% between 2015 and 2019, the European Region has almost reached an important milestone of the End TB Strategy (a 20% reduction by 2020) [8]. With a 31% reduction in deaths due to TB during the same period, the WHO European Region has also been on track to reach the 2020 milestone of a 30% reduction in the absolute number of deaths because of TB per year [3]. However, with an estimated 16–18% of all newly diagnosed patients and 45–59% of all previously treated patients having MDR-TB, the Region remains well above the global average of 2–4% and 10–27%, respectively [3]. This situation posed a risk to meeting the ambitious 2025 milestones of the End TB Strategy, particularly in the eastern part of the Region, already prior to the COVID-19 pandemic.

Since the beginning of 2020, the emergence of SARS-CoV-2 has had large health, social, and economic impacts. Two modelling analyses predicted that the annual number of deaths due to TB globally could rise to the levels last seen in 2015 or even 2012 [9,10]. First data released by the StopTB partnership indicate that, as of March 2021, TB services are still impaired in most high burden countries and that a total decline of 1 million cases being diagnosed with TB and enroled in treatment was observed in nine high burden countries that represent 60% of the global TB burden [11]. Provisional data collected by WHO from 84 countries indicate that an estimated 1.4 million fewer people received care for TB in 2020 than in 2019 [12]. Although additional analyses have been performed addressing the changes in TB notification rates since the onset of the COVID-19 pandemic and its worldwide effects on clinical TB services, few studies have investigated the impact of the pandemic on TB diagnostic services [13-16]. In a WHO survey conducted among 184 National Tuberculosis Programmes, 46% (85/184) respondents reported reallocation of staff at national or subnational level, 28% (52/184) reported reallocation of funding towards the COVID-19 response, and 23% (43/184) reported reallocation of GeneXpert PCR machines for SARS-CoV-2 testing. Notably, the corresponding proportions were considerably higher among a subgroup of 30 countries with particularly high TB burdens (67%, 47%, and 43%, respectively) [3].

Our study investigating the impact of the COVID-19 pandemic on TB reference diagnostic services in the WHO European Region found that the majority of the participating NRL reported declines in sample numbers sent for TB diagnostics since the onset of the COVID-19 pandemic. Reasons for these declines possibly comprise a country-dependent mixture between limited access to healthcare facilities (e.g. travel restrictions or reduction in the number of health facilities offering TB diagnostic and treatment services), reduced frequency of outpatient visits for treatment monitoring, and less transborder migration as compared with the pre-SARS-CoV-2 period. Whether COVID-19 lockdowns led to a decline in TB incidence or whether such lockdowns even promoted household transmission remains a matter of further research [17]. We also found that more than half of the participating laboratories performed SARS-CoV-2 testing in addition to their regular duties, mostly without additional staff ressources or additional funds for salary compensation. It should not go unnoticed that this has been a remarkable achievement.

This study has some limitations. First, the chosen study period did not cover the second and third COVID-19 waves in full, and personal communications with many of the participating laboratory directors indicate that these may have affected some laboratories even harder than the first wave of the pandemic [18]. Second, the study was limited to reference diagnostic services and did not address any COVID-19 related impact on more peripheral laboratories. Third, we did not address response bias, observation bias or recall bias potentially leading to under- or overreporting of changes in sample numbers, workload or compensation efforts. Finally, this survey relied exclusively on estimates by the laboratory leadership in order to facilitate a rapid assessment of the situation. Studies analysing actual sample numbers would be desirable to further objectivise the impact of the COVID-19 pandemic on TB diagnostic services in the Region.

## Conclusions

A growing body of evidence suggests that the collision of the COVID-19 pandemic with other global healthcare threats such as HIV, viral hepatitis, antimicrobial resistance and TB puts at risk many of the achievements made in the fight against these diseases during recent years. For TB, our data demonstrate that reference diagnostic services throughout the WHO European Region were strongly affected by the COVID-19 pandemic in terms of lower sample numbers, reagent shortages and by supporting the important need for SARS-CoV-2 testing through reallocation of ressources. With respect to the latter, decision makers at all levels will need to keep ensuring preparedness against novel pandemic diseases in public health laboratories by providing adequate staff capacity and infrastructures, and by uninterrupted procurement of essential consumables and reagents.
