# Authors’ correction for Euro Surveill. 2021;26(24)

**DOI:** 10.2807/1560-7917.ES.2021.26.30.210729c2

**Published:** 2021-07-29

**Authors:** Eurosurveillance editorial team

**Affiliations:** 1 European Centre for Disease Prevention and Control (ECDC), Stockholm, Sweden

On request of the authors, the affiliation of Askar Yedilbayev was changed from “Infectious Diseases, Faculty of Medicine, Imperial College London, London, United Kingdom” to “Regional Office for Europe, World Health Organization, Copenhagen, Denmark” in the article ‘Impact of the COVID-19 pandemic on tuberculosis national reference laboratory services in the WHO European Region, March to November 2020’ by Maurer et al., published on 17 June 2021. This change was made on 29 July 2021. 

